# Neurocognitive Functions in Children and Adolescents with Subclinical Hypothyroidism

**DOI:** 10.4274/Jcrpe.497

**Published:** 2012-03-08

**Authors:** Ayça Törel Ergür, Yasemen Taner, Evşen Ata, Efnan Melek, Emel Erdoğan Bakar, Tanzer Sancak

**Affiliations:** 1 Ufuk University Faculty of Medicine, Department of Pediatric Endocrinology, Ankara, Turkey; 2 Ufuk University Faculty of Medicine, Department of Child and Adolescent Psychiatry, Ankara, Turkey; 3 Ufuk University Faculty of Medicine, Department of Pediatrics, Ankara, Turkey; 4 Ufuk University Faculty of Medicine, Department of Psychology, Ankara, Turkey; 5 Ufuk University Faculty of Medicine, Department of Radiology, Ankara, Turkey; +90 312 204 42 90aycaergur@superonline.com

**Keywords:** hypothyroidism, neurocognitive functions

## Abstract

**Objective:** Hypothyroidism is a metabolic condition that can lead to cognitive and behavioral deficits in children and adolescents. However, there is less evidence about subclinical hypothyroidism (SH) as a risk factor for neuropsychological disorders in childhood. The aim of thisstudy was to evaluate cognitive functions like active/passive attention, maintaining attention, and response inhibition in pediatric patients with SH.

**Methods:** Seventeen patients (between 7-17 years old) with SH were tested with the Stroop test, Verbal Fluency test and the sub-tests of the Wechsler intelligence scale for children-Revised (WISC-R). SH diagnosis was based on the mild increase of serum thyrotropin (TSH) level together with a normal serum free thyroxine level and an exaggerated TSH response to thyrotropin-releasing hormone.

**Results:** Out of seventeen cases, 10 (59%) were girls and 7 (41%) were boys. Six cases were obese and 5 were overweight. The children in the SH group, as compared to the control group, obtained significantly lower scores on both the Digit Span subtest of the WISC-R and the Stroop subtests, which are sensitive to attention. No significant differences were found between the SH group and the healthy controls in verbal fluency and encoding tests.

**Conclusion:** In this study, pediatric patients with SH showed poor performance in tests measuring attention. Therefore, we want to stress the importance of close collaboration between pediatric endocrinology and child and adolescent psychiatry departments.

**Conflict of interest:**None declared.

## INTRODUCTION

The known effects of thyroid hormones on functions of the central nervous system include effects on intelligence, emotional status, behavior and cognitive functions (1,2). Hypothyroidism is a metabolic condition that can lead to cognitive and behavioral deficits in children and adolescents (3,4). Current data suggest that early diagnosis and treatment of hypothyroidism improves cognitive functions (5). It has been suggested that subclinical hypothyroidism (SH), characterized by mild increase in serum thyrotropin (TSH) together with a normal serum free thyroxine (fT4) level, is a risk factor for development of systemic diseases such as atherosclerosis, cardiovascular diseases as well as neuropsychiatric disorders. Panic attacks, depression, attention deficit, deterioration of memory have all been reported in SH as disorders which remain silent for long periods of time before their symptoms become apparent (6,7,8). 

With this current study, we aimed to evaluate cognitive functions such as active/passive attention, ability to maintain attention, and response inhibition in patients with SH.

## MATERIALS AND METHODS

The study sample consisted of 20 patients between 7-17 years of age, who had been referred to the Outpatient Clinic of the Department of Pediatric Endocrinology of Ufuk University School of Medicine in Ankara, Turkey with TSH elevation or with diverse complaints and who had been found to have elevated TSH levels during routine examinations. Patients with any kind of systemic disease and/or taking medications were excluded from the study. A thorough physical examination including pubertal staging and anthropometric measurements were performed in all cases. Exact chronological age, height, weight, standing height, measurements expressed as standard deviation (SD) scores, and body mass index [BMI: weight(kg)/height(meters)2] were recorded. Obesity was defined as having a BMI greater than the 95th percentile for age and sex (9). BMI reference curves for Turkish children were used for the evaluation of obesity (9). 17 patients accepted to participate in the study. 17 healthy children and adolescents constituted the control group. Hormone assays were done in the laboratory of the hospital for each subject. Diagnosis of SH was based on mild increase of TSH (5-25mIU/L) with a normal fT4 level. Thyrotropin-releasing hormone (TRH) test was also performed in all children. TSH response to TRH stimulation was considered to be normal when TSH levels were between 5-25 mIU/L. Values above 25 mIU/L were accepted as exaggerated and those below 5 mIU/L were accepted as suppressed (10). Thyroid antibodies (anti-TPO, anti-TG) and iodine level in urine were measured; bone age and thyroid ultrasonography were evaluated in all patients. Morning urine samples were collected from all patients to determine urinary iodine excretion. Iodine in urine was measured using the colorimetric method as recommended by the World Health Organization and the International Council for the Control of Iodine Deficiency Disorders (WHO-ICCIDD) (11). The prevalence of iodine deficiency was graded according to the WHO classification (11). Encoding and digital sequence tests including the Turkish form of the Stroop test (TBAG), verbal fluency, and the sub-tests of the Wechsler intelligence scale for children-revised (WISC-R) were applied to all 17 cases diagnosed as SH and to the 17 healthy controls. The study was approved by the ethics committee of the medical school. Written consent was obtained from all participants. 

**Neuropsychological Tests**

**[i]The Stroop Color-Word Interference Test:[/i]**

This test assesses the response inhibition and measures the ability to shift perceptual set in accordance with changing demands. It also measures the inhibition of a habitual behavior pattern and behaving in an unusual manner. Defects in these abilities result in lack of perseveration, stereotypic behaviors, and difficulty in controlling behavior. These functions are mainly controlled by the frontal lobes. Higher interference scores indicate poorer performance. The Stroop test also assesses the information processing rate, the parallel processing of attended and non-attended stimuli, as well as attention (12). This test has been adapted and standardized for the Turkish population (13).

**[i]The WISC-R Digit Span Subtest:[/i]**

For Digit Span Forward, the child repeats numbers in the same order as read aloud by the examiner. For Digit Span Backward, the child repeats numbers in reverse as presented aloud by the examiner. This subtest evaluates immediate auditory recall, freedom from distraction, attention, concentration and mental control. The reliability and validity studies of the Turkish form were conducted in 1978 (14).The WISC-R Coding Subtest: The child copies symbols that are paired with simple geometric shapes or numbers. Using a key, the child draws each symbol in its corresponding shape or box within a specified time limit. This subtest evaluates visual-motor coordination, motor and mental speed as well as inhibition. The reliability and validity studies of the Turkish form were conducted in 1978 (14). 

**[i]The Verbal Fluency Test:[/i]**

The Initial Letter Verbal Fluency Test (FAS) evaluates executive functions (principally, a frontal lobe function) and semantic memory stores (a temporal lobe function). In a timed test (one minute), the patient generates words beginning with the letters K, A and S (“Association” score). The sample was also exposed to a semantic category (animals) and the number of generated words was scored (“Animals” score). Finally, letter fluency was combined with category fluency in which the subject had to switch between words beginning with the letter ‘‘K’’ and names of animals (‘‘Categories’’ score). The norm study for the Turkish population was conducted by Bingol et al (15).

## STATISTICAL ANALYSIS

The Statistical Package for the Social Sciences (SPSS) version 15.0 was used to analyze the data. The effects of SH on the neuropsychological test/task scores in the patient group were evaluated with the t-test for independent samples.

## RESULTS

Out of 17 cases, 10 (59%) were girls and 7 (41%) were boys. Ten patients were in the pre-pubertal period, while 7 showed signs of puberty. No differences regarding age and BMI were found between the cases with SH and the control group (Table 1). Six cases were obese and 5 were overweight. Goitre was noticed in two patients (TV > +2SD). All cases were negative for thyroid antibodies. All subjects had normal urine iodine levels, except for one patient who had mild iodine deficiency. No significant differences regarding the scores for the Total Intelligence Section of the WISC-R were found between the study (=109.34) and control groups (=111.27). Analyses have shown that the group effect was significant for the WISC-R Digital Sequences subtest and the Stroop Test-TBAG form (ST2-corrected score, ST4 error score and ST5 error score). No significant differences were found between the SH group and the healthy controls in the verbal fluency and encoding tests (Table 2).

## DISCUSSION

In a study on adults by Correira et al (16) in 2009, SH was shown to cause more deterioration of cognitive functions as compared to overt hypothyroidism. There are very few studies investigating the cognitive functions in children and adolescents diagnosed with SH. In the study by Aijaz et al (7), attention deficit was found to be more frequent in children diagnosed with SH than in healthy controls. Wu et al (2) reported that adolescents with a diagnosis of SH showed lower performance in tests measuring cognitive functions as compared to adolescents with subclinical hyperthyroidism. Conflicting results were obtained in studies measuring the outcome of neurocognitive functions in hypothyroid patients who were diagnosed during screening studies and treated at an early phase. In a meta-analysis including 7 studies, presence of intelligence quotient deficits was found in patients with congenital hypothyroidism despite early diagnosis and treatment (17). Aijaz et al (7) reported that T4 treatment did not exert a significant effect on the neurocognitive performance of patients with SH. 

In our study, no differences were found between the SH patients and the control group in level of intelligence. On the other hand, the children in the SH group, as compared to the control group, scored significantly lower on both the Digit Span subtest of the WISC-R and the Stroop subtests, which are sensitive to attention. Hypothyroidism is known to cause attention deficit problems in children (1). Attention deficit and hyperactivity disorder (ADHD) can be seen in children with thyroid hormone resistant hypothyroidism (18). Although the cases with SH in our study did not meet the criteria for ADHD, they showed poorer performance in tests measuring attention when compared to controls. 

In humans, a way of accessing the information maintained in precognitive sensory records to the consciousness (i.e. to short-term memory/working memory (STM/WM)) is through the effective attention they are given (19). However, new or sudden stimuli or stimuli which are important in terms of the species or the individual passively attract the attention. These stimuli also reach consciousness, just like those stimuli to which attention is actively given. Furthermore, information in the long-term memory can divert the direction of the attention, and selective attention can determine what will reach the STM by passing the data though the sieve of attention. Therefore, deterioration experienced in the area of attention negatively affects the STM performance in turn (19). With the purpose of predicting the difficulties in the areas of active/passive attention and the selective attention of the children in the study, WISC-R Digit Sequences Subtest scores were evaluated, and it was observed that the children diagnosed with SH obtained poor results in this subtest. The Stroop test demonstrates the ease of changing the perceptual organization according to the changing demands and under the influence of a “deteriorating effect”, the capability of suppressing a habitual behavioral pattern and performing unordinary acts (20). 

In the present study, it was observed that the cases diagnosed with SH, as compared to the control group, obtained higher scores particularly in the sections of the Stroop test regarding the maintaining of attention and controlling the instinctive responses. It is thought that these results originate from the difficulties these cases have in focusing their attention and in selecting the relevant cognitive activities. These individuals also have difficulty in suppressing information which is irrelevant. The lack of significant differences between the scores of patients diagnosed with SH and those of healthy controls in verbal fluency and encoding tests may have been due to the smallness of our sample. To demonstrate the cognitive changes in SH, it would be advisable to conduct these types of studies on larger groups of patients.

The findings of this study on SH, a condition that can impair attention, also remind us of the importance of collaboration between pediatric endocrinology and child and adolescent psychiatry departments. In summary, while no significant differences were observed between the groups in the tests and subtests evaluating the managing functions, significant differences were observed in neurocognitive tests and subtests measuring attention.

The limitation of this study is the relative smallness of the sample. However, as the studies in this field are very limited in number, we consider our work to be an important preliminary study. Another limitation of this study is absence of follow-up results after T4 therapy. This is also an important area of research as conflicting results have been obtained till now. Further studies are needed to elucidate many questions about these issues. 

## Figures and Tables

**Tab le2:**
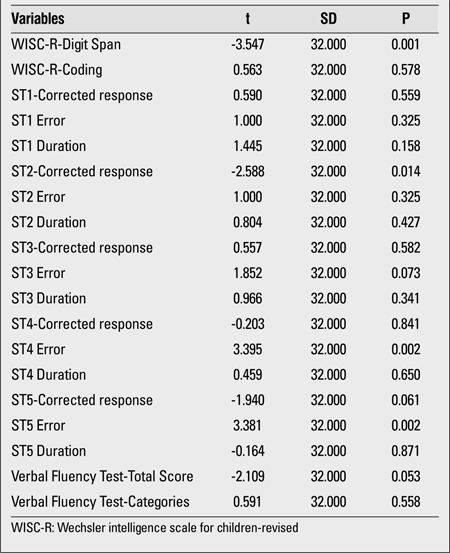
Neuropsychological test scores in children with subclinicalhypothyroidism and in the control group

**Table 1:**
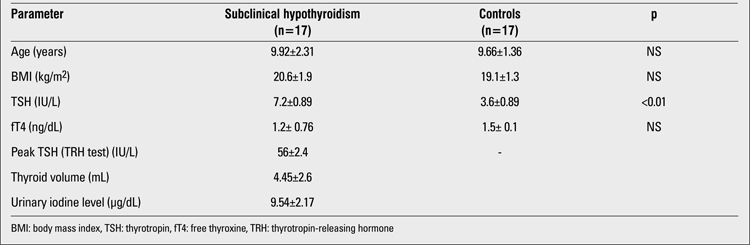
Mean age, BMI and thyroid function in children with subclinical hypothyroidism and in the control group
